# Understanding the Roles of Remoteness and Indigenous Status in Rural and Remote Road Trauma in North Queensland: Using a Mixed-Methods Approach

**DOI:** 10.3390/ijerph17051467

**Published:** 2020-02-25

**Authors:** Colin Edmonston, Victor Siskind, Mary Sheehan

**Affiliations:** 1 Queensland Department of Transport and Main Roads, Queensland University of Technology; Rockhampton, QLD 4700, Australia; Colin.J.Edmonston@tmr.qld.gov.au; 2 Centre for Accident Research & Road Safety-Queensland, Queensland University of Technology, Kelvin Grove, QLD 4059, Australia; m.sheehan@qut.edu.au

**Keywords:** Indigenous, distal causation, proximal causation, rural and remote crashes, road safety, transport disadvantage

## Abstract

Road trauma is a significant health problem in rural and remote regions of Australia, particularly for Indigenous communities. This study aims to identify and compare the circumstances leading to (proximal causation) and social determinants of (distal causation) crashes of Indigenous and non-Indigenous people in these regions and their relation to remoteness. This is a topic seriously under-researched in Australia. Modelled on an earlier study, 229 persons injured in crashes were recruited from local health facilities in rural and remote North Queensland and interviewed, mainly by telephone, according to a fixed protocol which included a detailed narrative of the circumstances of the crash. A qualitative analysis of these narratives identified several core themes, further explored statistically in this sample, supplemented by participants in the earlier study with compatible questionnaire data, designed to determine which factors were more closely associated with Indigenous status and which with remoteness. Indigenous participants were less often vehicle controllers, more likely to have recently been a drink driver or passenger thereof; to be unemployed, unlicensed, distracted or fatigued before the crash, alcohol dependent and have lower perceived social, but not personal, locus of control in a traffic crash than non-Indigenous persons. Differences between Indigenous and non-Indigenous participants are largely ascribable to hardship and transport disadvantage due to lack of access to licensing and associated limitations on employment opportunities. Based on these findings, a number of policy recommendations relating to educational, enforcement and engineering issues have been made.

## 1. Introduction

Road trauma resulting from vehicular crashes has been a longstanding cause of death and injury in rural areas of Australia and, like other causes of injury, the risk increases with remoteness [1]. This, and the sequelae of such trauma, places a significant burden on health services in rural areas which tend to be under-resourced in terms of health care personnel, specialised skills and capacity [2]. An issue that remains understudied and uncertain is the relative contribution of rural factors including road characteristics and related personal driving styles to high levels of crashes or are these characteristics more likely to be experienced in a remote region? This question has particular relevance to understanding the comparatively high road fatality and serious injury rates of Australia’s Indigenous people who disproportionately live in the more remote areas of the country.

Indigenous people have been identified as a particularly high-risk population [3]. Despite the data limitations plaguing Indigenous research, it is widely accepted that Indigenous Australians are two to three times more likely to be killed and 1.3 times more likely to be hospitalised due to a land transport crash than their non-Indigenous counterparts; Indigenous pedestrians are particularly at higher risk [4,5]. While this over representation may partly reflect the larger proportions of Indigenous Australians living in remote areas disparities in both the scope and nature of Indigenous versus non-Indigenous crash profiles in the rural setting remain [6]. For example, Indigenous people are significantly more likely to be seriously injured as passengers or pedestrians, while non-Indigenous people are more likely to be seriously injured as drivers or motorcycle riders [7].

The overrepresentation of Indigenous Australians in road trauma and common crash types are consistent with the First Nations experience in New Zealand, United States and Canada [8,9] (pp. 45–59). Improvements over time in the New Zealand (Māori) context has potential to inform the way Indigenous road safety is delivered in Australia. 

There is a growing body of research to suggest that the crash profile associated with road trauma in Indigenous communities is related to an array of social and environmental factors including ‘transport disadvantage’ [6,10], reduced access to licensing programs and culturally suitable road safety education [11,12,13,14] and reluctance to fully utilise organised health and rehabilitation services [15,16]. The need to develop appropriate and accessible pathways to obtaining and maintaining a driver’s licence in Indigenous communities, with related road safety benefits, is gaining national momentum [17,18,19].

To further explore the causal elements of road trauma in the rural and remote context, a major *Rural and Remote Road Safety Study* (RRRSS) was undertaken in which interviews were conducted in hospitals with persons injured in a vehicular crash, both on and off road [20,21]. Crashes occurring in the two major urban areas in the region were excluded. An important component of the interview was a ’crash narrative’ section in which participants described the circumstances of their crashes. This was accompanied by a detailed questionnaire which sought information on demographic variables, substance use, past experience of crashes and drink driving, trip and vehicle characteristics, and attitudinal and personal details. Interestingly, this study found that after controlling for human factors, vehicle and road conditions, made a minimal contribution to the seriousness of the crash outcome. As such, the primary focus of our program of research was exploring human factors requiring attention, most notably the ‘Fatal 5’ [22] and Indigenous status. 

While the narrative-based methodology (story-telling approach) aligns well with Indigenous culture, there were too few Indigenous participants in this study to make comparisons by ethnicity. This paper includes additional reports from a follow-up study with a modified research design and recruitment strategy to boost the number of Indigenous interviewees [9]. It was designed to profile the unique and shared characteristics of crashes involving Indigenous people compared to other rural and remote road users through an analysis of patients’ crash experiences.

With an end goal of improving Indigenous road safety policy and delivery in Australia, the primary aim of this study was to identify which behavioural, environmental and vehicular crash risk factors are more closely associated with Indigenous status and which with remoteness. It was hoped that a better understanding of the circumstances of crashes in rural and remote areas (proximal causation) and any attitudes and social factors associated with these behavioural characteristics (distal causation) could inform this end goal. Research by Fitts and her colleagues with an offender population demonstrated that attitudes of hopelessness and bravado influenced the propensity of Indigenous people to engage in drink driving behaviour [23]. This paper examines all crash types to further explore the roles that attitudes and social factors play in influencing risk taking more generally and identifies opportunities to use these findings to inform interventions. 

Initially, this paper reports the findings of a qualitative analysis of the patient crash narratives. The results document a thematic analysis of contributing factors (proximal causation) and social determinants (distal causation). Key themes—’variables of interest’—identified through this process are further explored in a quantitative analysis using questionnaire data from both the sample recruited by Edmonston and that by Sheehan et al. [9,20]. This paper brings together the findings of both analyses to discuss at length the implications for Indigenous road safety policy and practice. 

## 2. Methods

While the RRRSS was conducted solely in four large hospitals in North Queensland and interviewed only persons admitted for at least 24 hours, the follow-up study extended recruitment to smaller health facilities throughout the Northern, Far North and North-West Statistical Divisions of Queensland. After approval had been obtained from all relevant Research Ethics Committees, all local health facilities and outpatient clinics were approached and invited to participate in the recruitment process. Ideally, every person over the age of 16 years presenting to one of the recruiting health facilities with injuries incurred through a road crash whether as controller, passenger, cyclist or pedestrian and whether admitted or not, was to be approached to take part. The purpose of the research was explained by health service staff and the person given the opportunity to contact the principal researcher (CE) via telephone on a free-call number. As an incentive, a payment of A$20 was offered for a completed interview, to be paid by a nurse at the health facility on receipt of confirmation from the researcher. The protocol ensured that anonymity of the participants was preserved. A detailed description of the recruitment procedure is provided in the full report, including efforts made to train nursing staff in participating facilities and to maintain their continued participation [9] (Chapter 4). At the same time, recruitment continued at two larger hospitals in the region using similar procedures as in the RRRSS, but with less emphasis on length of stay. As in RRRSS, crashes occurring in urban areas were out of scope. The data collection period ran from February 2007 to August 2008 but varied slightly across sites.

The interview protocol used in this study was adapted from that used in the RRRSS to maximise understanding among Indigenous and remote non-Indigenous populations. Its development was overseen by an Indigenous Reference Group [9] (pp.67) who ensured all aspects of the research were conducted in accordance with the six core principles underpinning ethical research in Aboriginal and Torres Strait Islander communities [24]. The interview protocol relied heavily on the crash narrative in response to the question, “I realise that this might bring back some difficult memories, but I was wondering if you could tell me a little about what happened before, during and after your crash and what you think may have caused it?”. Relevant prompts to align with the Indigenous familiarity with a story-telling approach were used [25]. The modified instrument replaced the previous Likert-type response scales with pictorial items, for example circles of different sizes, to better accommodate the unfamiliarity of some Indigenous cultures with abstract numerical concepts [9] (pp.84). Apart from the crash narrative, topics included were background and demographics; driving experience; possible risky or illegal behaviour prior to the crash (e.g., being unlicensed, alcohol or drug use, speeding); use of protective equipment (seatbelts, helmets); trip characteristics (e.g., purpose and duration of journey, periods of fatigue, monotony, distractions); vehicle characteristics and maintenance; self-reported prior crashes and traffic offences; individual and community road safety attitudes and practices, including locus of control, enforcement and specific countermeasures; regular alcohol use as measured by the AUDIT-C scale [26]. Efforts were made to ensure information collected from the additional cases recruited through the study was comparable to information collected in the RRRSS. The questionnaires, differing slightly for each type of road user, may be viewed at [27]. Interview length ranged from 35 minutes to 2 hours and 40 minutes in duration. 

Participants self-identified as Indigenous or non-Indigenous. Geographic comparisons were based on crash location for factors directly related to the crash or place of residence in the case of factors unrelated or only indirectly related to it. The modified Accessibility/Remoteness Index of Australia (ARIA+), a five-point scale ranging from Metropolitan to Remote, was used to distinguish between rural and remote localities [28,29].

For the qualitative part of the study, a thematic analysis of the content of narratives was conducted by the primary researcher (CE). In terms of process, individual narratives were ‘indexed to generate or develop analytical categories and theoretical explanations’ as they relate to the distal and proximal causation of crashes described by the sample [30]. Then, using a Grounded Theory approach, a ‘constant comparison method’ was used by both the primary researcher and a research assistant to identify similarities and differences across units of data/groups [31]. In addition, 10 percent of cases were randomly reviewed by members of the project team to verify themes. In the quantitative analysis, most dependent variables considered were dichotomous or had been recoded as such. For these the analytic approach employed was logistic regression analysis to determine the individual associations with Indigenous status and remoteness as well as any interaction effects. In the logistic analysis, each appropriate dichotomous variable in turn is the dependent variable, while Indigenous status, remoteness and their interaction form the predictor variables. The sole ordinal variable subjected to analysis was the AUDIT-C scale, a measure of alcohol use and dependence [26], for which two-way analysis of variance was used. Proportions of harmful drinking, as defined by the scale, were also calculated. The purpose of the analysis is only to determine whether an association exists beyond chance, its direction and an indication of its strength rather than to estimate design parameters. Since risk factors for traffic crashes are well established, the present analyses are not designed to explore them further.

In order to increase the power of the analysis, the sample was supplemented with 34 Indigenous and 357 non-Indigenous persons who had participated in the earlier RRRSS study in all analyses in which the included variables were common to both samples.

Results of the logistic regression analysis are given as Wald chi square, with one degree of freedom, significance level (*p*) and the odds ratio (to one decimal place) as a measure of strength of the association with Indigenous status and with remoteness, with the interaction term being included if statistically significant or marginally so. The method of data collection does not warrant greater precision, although, as discussed later, is unlikely to lead to significant bias. Analyses were conducted using Statistical Package for Social Sciences, Version 18.0 (SPSS, Armonk, NY, USA) software. It should be noted that the numbers of individuals in a given analysis will vary depending on applicability and the availability of information. 

## 3. Results

### 3.1. Qualitative Analysis

Recruitment into the supplementary sample was facilitated through two hospitals and 31 local health facilities, resulting in 80 Indigenous and 149 non-Indigenous participants. Using the qualitative analysis techniques described above, several themes were extracted. These themes have been grouped below according to their nature, either as describing contributing factors (proximal causation) or social determinants (distal causation). 

#### 3.1.1. Thematic Analysis of Personal Proximal Contributing Factors 

##### Externalisation of Blame: Alcohol or the Road?

The major point of difference between the Indigenous and non-Indigenous participants was an inclination to externalise responsibility for the crash. Indigenous respondents were more likely to admit to their role in the crash, including alcohol involvement, whereas non-Indigenous respondents tended to blame external factors or “bad luck”. Typical comments were:

*“I was walking down … Street just after dark and a 4WD hit me. Lucky he was going slow. The mirror on the car hit me and I got a sore shoulder. I know the driver and he was sorry but I was pissed off. He shouldn’t be driving—he doesn’t have a licence and was drunk*.

(Remote, Indigenous, Pedestrian)

*“I was running across the road to cross to the other side and got hit. I saw the car but he seemed a long way away … I don’t remember getting hit but I just remember waking up in a pool of blood. I was drunk, that’s why it happened.”*.

(Rural, Indigenous, Pedestrian)

*“I’d been drinking grog all day—‘goon’ (wine). I drink it most days. I was heading back … towards home and drifted off the road. I think I was passing out from all the grog. I hit some of the posts marking the roads and hit the brakes pretty hard. Next thing I hit a tree. Blacked out after this. The community police woke me up … Everything was blurry … They made me blow into the bag. I knew I was over. I will probably lose my licence for more time now but I’ll learn my lesson one day”*.

(Rural, Indigenous, Driver)

The narratives provided support for the contention that alcohol use among Indigenous people is bimodal, with a tendency towards abstinence or excessive use [24]. When alcohol was a factor, drivers were more likely to adjust other behaviours, such as speeding to compensate for being intoxicated.

*“Was a real bad smash mate—real bad. We’d been at the pub all morning … There were six of us and we were drunk on grog. Drunk the night before too—it’s what we do. About half way home the driver lost it on a bend. We were flying—maybe going 150km/h, maybe quicker. He turned sharp but it was too late—the car flipped and rolled. We were thrown around but stayed inside the car. We crawled out of the car and pushed it back on its wheels. Nothing was broken [bones]. It took ages for the ambulance to come … The speed is what caused us to crash. If you’ve been on the grog you need to slow right down”*.

(Remote, Indigenous, Passenger)

Non-Indigenous participants were more like to externalise the crash to the condition of the road or distractions, even when reporting having engaged in risky behaviour.

*“This crash wasn’t my fault. I’m a truckie, so the highway is my office. I’m always safe and wear all the safety gear on my bike as I did Sunday. I was heading to …. I went to go around a bend … and lost control because there was gravel left on the road. They [Main Roads] didn’t clean up after roadworks. It was a right hand turn and maybe I was going a bit fast for the corner”*.

(Rural, Non-Indigenous, Rider)

*“It was right on dusk … I saw a roo on the road and swerved slightly. I caught the loose gravel on the edge of the curve and slid into a tree … I had a few drinks earlier which may have affected my reflexes but the roo really caused me to crash. I was only going about 70km/h but that is as fast as you can go on the track, it’s pretty slippery”*.

(Rural, Non-Indigenous, Driver)

*“I was leaning out of the car with the door open—putting down some markers for the Royal Flying Doctor Service. My mate was driving, only going slow. He was distracted by a roo and swerved sharp … Bugger the roos, they’re everywhere out here and caused our crash”*.

(Remote, Non-Indigenous, Passenger)

*“Another example of car drivers not seeing motorbikes. This car just pulled out in front of me. He didn’t even look. … I was going fairly quick, not expecting him to pull out. I saw him and tried to stop but it was too late”*.

(Rural, Non-Indigenous, Rider)

##### Distraction: “I only Looked away for a Split Second”.

Distraction was the most commonly cited factor among all patients. In total, 35.4% of cases reported it as part of a causal chain, where it was more prevalent in remote (53.8%) than rural (25.5%) crashes. Further, the nature of the distraction was contextual to the crash setting.

*“Just as I was turning I heard my phone. I looked down to see who text me and missed the bend. The car rolled into a gully—my life flashing before my eyes. I think I had shock or concussion because I don’t remember much after the crash … I shouldn’t have played with my phone and got a cab home”*.

(Rural, Non-Indigenous, Driver)

*“Bloody rear-ender at the lights. I was checking my phone and didn’t stop in time. It happens”*.

(Rural, Non-Indigenous, Driver)

*“Heading home from shopping…. Just bought the kids (hamburgers) and they were fighting in the back. I turned around to sort it out and run off road. I was distracted by the kids and struggling with the car”*.

(Rural, Non-Indigenous, Driver)

Common external distractions, particularly in remote settings, were, for instance, animals, looking at friends or scenery. Another commonly mentioned factor was inattention due to a recent incident or emotional event (i.e., domestic dispute). Inattention was often associated with alcohol use.

*“I looked away for a second or two at the water. When I looked up I saw a car coming and swerved. I must have hit a pothole or something because the next thing I knew I went straight over the handlebars—my stomach hit the handlebars hard and then I fell on the ground … I wasn’t wearing a helmet but I didn’t hit my head”*.

(Remote, Indigenous, Cyclist)

*“It’s very dark on that road and more than half way home I saw a cow in the middle of the road. She wasn’t going anywhere and I knew I couldn’t stop. I was taught not to swerve to miss animals so I hit it. My car was a write off but I’ll live … I can’t say the same for the cow”*.

(Rural, Non-Indigenous, Driver)

*“Family problems and been drinking the night before … I was driving to work—almost there. I was thinking about the fight I had the night before … Ran off the road and hit a tree … I travel that road every day and was on automatic pilot”*.

(Rural, Indigenous, Driver)

##### Not Wearing Personal Protective Equipment: “Not Going far”

Another common theme, particularly in remote settings, was the lack of use of personal protective equipment (PPE), such as restraints or helmets. Participants often recognised the necessity of PPE during and following a crash, but due to a variety of reasons such as lack of enforcement, not being available, or impaired decision making due to intoxication, it was not used. Participants often reported balancing the lack of PPE with another safe practice.

*“If we’re not going far we don’t worry … You usually won’t get caught and you’re going slow—it’s safe. I suppose if the driver’s drunk you should wear your seatbelt to balance it out”*.

(Remote, Indigenous, Passenger)

*“We’d been drinking most of the day at the footy and were on our way home. We probably should have just walked—taken the foot Falcon—because we only had a little way to go. We didn’t have our belts on either—seemed silly to put them on for a couple of streets. You just don’t know when you’ll need them I guess”*.

(Remote, Indigenous, Passenger)

*“I wasn’t wearing a helmet and only had one shoe on—the one I need to start the bike. I know you should wear it, but it’s not illegal [off road] … I was having a crack at a 12 foot jump and got it all wrong. Not experienced enough … I’ll get it right next time”*.

(Rural, Non-Indigenous, Rider)

The following key quotes below suggest that riding in the back of utes (“pick-up trucks”) is a common practice (despite the illegality) for both Indigenous and non-Indigenous persons in remote North Queensland.

*“A whole crew of us were piled into the work ute heading back … I felt the ute slip and slide around underneath me. I had hit some loose gravel. I should have tried to steer out of it but I panicked and hit the brakes. A couple of boys in the tray were thrown out. One flew through the air like a rag doll. I felt real bad, because I should have handled it better. I know to slow down a bit there—I drive it all the time with the guys … You never see any cops”*.

(Remote, Non-Indigenous, Driver)

*“I was running the council ute over to the workshop—it was smoko time. A dog ran out and I had to jump on the brakes pretty hard. One fella in the back of the ute fell out. He had cuts on his legs and couldn’t walk real good … I wasn’t going faster than the speed limit—dogs run out on the road all the time. I don’t have no licence but I’m going to get one. I hope the cops go easy on me”*.

(Remote, Indigenous, Driver)

*“We were walking back from the pub. There were about six of us. Our mate pulled up in his ute and we all jumped in the tray-back and he rolled it on the next corner … He may have taken the corner a bit fast … I know it’s illegal but we were weren’t going far”*.

(Remote, Non-Indigenous, Passenger)

##### Inappropriate Speed: “You Can Go Quick Out Here”

Another predominant theme in the interviews was speed, as it featured in crash descriptions by both patient groups. In rural areas, it was cited as “approaching an intersection too fast”, resulting in give way issues, or exceeding the speed limit. For remote areas it was related to the environment, as vehicles were “flying” (exceeding the rural limit of 100km/h) or “going too fast for conditions”.

*“Speeding brother caused our crash. The road was not too good ‘cause of the wet season. The driver lost control on a corner. He was going too fast for them roads … There was six of us in the car. We left the car there. There’s wrecks all along that road. Lots of fellas have crashed along there before”*.

(Remote, Indigenous, Passenger)

*“Badly running late for work … I was flying along—maybe 130km/h or so. I felt the car start to slide and couldn’t get it right. The car hit a cement drain and flipped … I never told the cops just how fast I was going. I drive along that road every day. I should know that bend like the back of my hand”*.

(Remote, Non-Indigenous, Driver)

*“It was Sunday arvo and I was heading out … for a ride. I saw some cars coming up behind me so I decided to give it some. I couldn’t hold it together going around a bend and crossed the centre lines. There was a car coming the other way so I had to head bush to miss him. I fell off down a gully and landed on my left arm … I was speeding like a dickhead so shit happens. I should have just chilled”*

(Rural, Non-Indigenous, Rider)

Comments about exceeding the rural limit were often qualified by statements about a lack of signage or safe speed cues: *“You can go quick out here*—*nothing tells you otherwise”* or the unlikelihood of detection—*“There’s never any cops on that road” … “Who’s going to catch me?”*

##### Fatigue: “Tired from All the Drinking”

Fatigue was more common among Indigenous road crash participants and was typically identified as a by-product of excessive alcohol consumption the night or day before.

*“It was early in the morning … He was half asleep because we’d been drinking the night before and going too fast for the corner—you need to slow right down there … I was just happy to live. The cops are going to throw the book at my mate—they know him”*.

(Remote, Indigenous, Passenger)

*“I’d been up all night. I don’t know how many … heavies [full strength beers] I had—more than a carton maybe. It was morning and I was driving home … I must have gone to sleep—real tired from all the drinking—because when I woke up I’d smashed into a power pole and the car was on its side. You shouldn’t drive after that much grog”*.

(Remote, Indigenous, Driver)

The following quote illustrates the impact of circadian rhythms on alertness levels.

*“I was feeling tired. Black fellas get tired after a feed. It’s like ‘goanna syndrome’—you have lunch and you want to have a camp in the sun [patient laughed]. I parked on top of a hill. I thought I put the car in park but maybe not …”*.

(Remote, Indigenous, Driver)

##### Unlicensed: “Nobody to Teach Me the Rules”

An important theme identified was the issue of training and education of rural and remote, particularly Indigenous, persons, on obtaining a license, and road safety. The key quotes below illustrate the many challenges and consequences that remain.

*“I didn’t do much school, so I can’t read and write good and don’t have a licence but I still need to get around. There’s lots of fellas like me”*.

(Remote, Indigenous, Driver)

*“The ‘bolliman’ [policeman] came to the clinic not long after [my crash] and knew that I’d been drinking and didn’t have a licence. They said that I could deal with it later [when] I was fixed up. They [clinic staff] flew me to Cairns to get my head checked out later that arvo”*.

(Remote, Indigenous, Driver)

*“I’d love to get a licence but what hope have I got—there’s nobody to teach me the rules. The coppers don’t want to know … We need help. You end up driving and getting caught”*.

(Remote, Indigenous, Driver)

Very few non-Indigenous participants disclosed being unlicensed drivers. Those who did were “once-a-week motor cycle riders”, holding car licences only, and occasionally rode on gazetted roads to access off-road locations. 

##### Crash Prone: “Talk about Bad Luck”

The last theme regarding contributing factors that emerged from the transcripts related to attitudinal challenges for rural road safety practitioners. A small number of participants stated that they were “just unlucky”, then explaining to the researcher that they are “really careful”. It was difficult to understand these perceptions as they described a continuous pattern of risk taking. Further review of their narratives supported the aphorism that ‘people drive as they live’ [31]. In addition, alcohol was a common theme in their accidents.

*“I’m having a bad run. Hit my head in a pool accident on Thursday and got king hit at the pub on Friday … Drank all over the weekend and crashed Monday … I was over the limit and going about 30k over … But I’m more careful than most around here”*.

(Rural, Non-Indigenous, Driver)

*“Smoked pot the night before and only had four hours sleep … Never wanted to ride bikes but here I am … I’ve written off five cars and four bikes in my life and lost my leg in my early 20s … I crashed again and was here three weeks ago … As a rule, I’m fairly careful and much safer now that I have kids”*.

(Rural, Non-Indigenous, Rider)

*“I crashed twice in two days. I’m riding up to the tip … The first day I lost control in a washout and fell off because the bike was overloaded with stuff … I decided not to do your study then … The next day I felt good enough to ride on and hit a cow … Talk about bad luck … Anyway, I thought I’d better tell you my story now … I was going about 80km/h—probably a bit quick both times”*.

(Remote, Non-Indigenous, Rider)

Consistent with the findings of the larger Rural & Remote Road Study [22] the ‘Fatal 5’ (speed, alcohol, fatigue, no restraints and distraction) figured prominently for both groups with at least one of these core behaviours identified by more than 90 percent of patients. Further, using patient narratives, the next section explores some of the social context and attitudes (distal causation) associated with these behaviours (proximal causation).

#### 3.1.2. Thematic Analysis of Social Distal Determinants

##### Rural Rituals: “Do It All the Time and Nothing Bad Happens”.

The following themes illustrate the rural way of life, in which the pub (public house or licensed hotel) is an important social centre. Due to reduced exposure to enforcement and shared acceptance of risk, this way of life reflects poor public transport options, decreased seatbelt compliance, and higher travelling speeds. Participants’ accounts also imply that these behaviours are ritualistic, as they engage in risky behaviours on a daily or weekly basis because crashes are relatively rare and safety concerns are low. The following key quotes from both Indigenous and non-Indigenous participants illustrate the power of the attitude that individuals can regularly take part in risky behaviours without concern or fear of negative consequences. As some participants do not address this behaviour even following multiple crashes, it is therefore imperative that addressing ‘this rural way of life’ becomes a road safety priority.

*“Heading home from the pub after happy hour on Friday. Only had to go a few blocks. My mate tried to turn into … Drive and stuffed up. Instead we ended up hitting a concrete sign … We were having a good night. We do the same thing every week”*.

(Rural, Non-Indigenous, Passenger)

*“It’s just the way it is out here … We all do the same things. We risk it with drink driving, speeding and overloading because we’re not likely to get caught and don’t have much choice. You have to get around and we have less options … If the roads were better though, there wouldn’t be as many crashes”*.

(Remote, Non-Indigenous, Rider)

*“It wouldn’t have been so bad if they weren’t piled in the back of the tray but that’s what happens out here. You all pile in. It’s no big deal—everybody does it and the cops don’t worry. Nothing ever goes wrong—except for the other day [patient laughed a little]”*.

(Remote, Indigenous, Passenger)

*“I travel this road all the time … on automatic pilot … You don’t think you’ll be the one in an accident”*.

(Rural, Indigenous, Driver)

*“I’d got on the drink with the boys after work. We always have Friday arvo drinks. I was feeling a bit pissed so I thought I’d better go home and drop the bike off before going back out to the … Hotel. I usually ride home after a few and nothing happens. I take it easy. You don’t think you’ll crash and I don’t know how it happened—I just lost it. I’m only on my Learners so I’ll probably have to go to court”*.

(Rural, Non-Indigenous, Rider)

*“It happened during my weekly Sunday ride. The bike got caught in a gravel dip and I fell off … It was my favourite time of the week and I was starting to be a pretty good rider I think. I haven’t been riding that long—only a couple of years”*.

(Rural, Non-Indigenous, Rider)

*“My partner was fiddling with the radio. I said ‘leave it’ and we had a bit of a spat—next thing we were heading off the road. … We were going too fast for the unexpected. We slammed into the tree pretty hard … I was lucky I had my seatbelt on or it could have been a different story. We’d been on the rumbos so I knew the cops would get (the driver) for drink driving. He was drunker than me. We were just unlucky—this is part of our ritual and usually nothing goes wrong”*.

(Remote, Non-Indigenous, Passenger)

##### Being a ‘Hero’: “Showing Off in Front of My Mates”

Several participants in the current sample attributed their risk-taking behaviour to bravado mentality, such as by seeking approval from their friends by “being a hero”. In one example, this involved falsely claiming responsibility for a crash to help a friend. These behaviours mirror what has been reported in interviews in a separate study with 73 Indigenous drink drivers [24].

*“We smashed into a tree when we were doing ‘burn outs’ …. We go there a fair bit to get away from the cops. They took my mate’s driver’s licence off him and me too. It’s part of becoming a man around here … We had a fair bit of grog and some gunga (marijuana) but we can handle it”*.

(Remote, Indigenous, Passenger)

*“I was trying to be a hero … Showing off is part of … life. We were taken turns at strapping [speeding and fishtailing] in this car we stole … The cops came when we crashed it. My mate was driving then but I said I was … He’s got more to lose. He would have gone to jail”*.

(Remote, Indigenous, Passenger)

This theme was present among both groups in the sample, indicating that intentional risk-taking, or as one participant put it, “pushing boundaries” is probably part of the young rural male rite of passage.

*“There were five of us and we were just doing some rough trail riding for fun … We were racing—trying to outdo each other. I looked back to check on the bloke behind me and when I turned back towards the front I didn’t have time to avoid this branch”*.

(Rural, Non-Indigenous, Rider)

*“It’s about who can do the biggest jump—being a hero … I’ve done it lots of times before and familiar with the track but I just didn’t give it enough revs … I’ll get it right next time”*.

(Rural, Non-Indigenous, Rider)

##### Hopelessness: “I Don’t Care What Happens to Me”

The following key quotes highlight the cultural and contextual framework of the behaviours and attitudes of Indigenous crash participants. They are influenced by life circumstances (importantly, employment status, hardship and transport disadvantage) and community-based beliefs about lack of control over one’s fate. The following quotes build a narrative exploring this relationship and reinforce the important symptomatic role of excessive alcohol use. This is an important theme as it feeds into the discussion of ways to improve Indigenous road safety.

*“You’ve got to get your family places and there’s not many cars … We were piled in [overloaded and unrestrained] like usual … What other option do we have”*.

(Remote, Indigenous, Passenger)

*“My mate [the driver] was drunk and has no licence … He might go to jail this time which will be real bad for his kids … It’s just what happens when you don’t have a job and nothing to do”*.

(Remote, Indigenous, Passenger)

Patterns of alcohol use in Indigenous communities is commonly bimodal [24], as individuals either drink at a harmful level or abstain.

*“I was walking down … Street just after dark and a 4WD hit me. Lucky he was going slow. The mirror on the car hit me and I got a sore shoulder. I know the driver and he was sorry but I was pissed off. He shouldn’t be driving—he doesn’t have a licence and was drunk. I don’t like drinking—some people don’t at all and others drink way too much. That’s communities—there’s no in between … He should have got his missus to drive, she doesn’t drink … Fellas don’t like women driving—it’s a power thing”*.

(Remote, Indigenous, Pedestrian)

*“I was in labour and my boyfriend was driving me to the hospital. I didn’t want him to drive me because he was drunk and he doesn’t have a licence but he just took the keys off his uncle … He had options but he has to drive. We were nearly there when we went over a hill and saw a car coming the other way. I’m not sure what happened but we hit it. We were going too fast for that road and I think they were too … My boyfriend broke his arm but I was more worried about the baby. I thought she would die before she was even born. It was a miracle that she was born ok. I would never have forgiven my boyfriend if our baby died”*.

(Remote, Indigenous Passenger)

In summary, patient narratives exposed three broad themes providing insights into the decision-making underpinning common crash profiles and risky behaviours. Two of the themes—termed ‘rural rituals’ and ‘being a hero’—were espoused by both Indigenous and non-Indigenous patients. The first theme supports the notion [32] of a ‘rural way of life’ in which patients regularly engaged in risky behaviour because of a lack of transport options, reduced exposure to enforcement and a shared acceptance of risk [32]. The strength of this theme was illustrated through many narratives and often highlighted the central role that alcohol and the local pub or licensed drinking establishment plays in rural areas. The second theme showed both Indigenous and non-Indigenous patients engaging in intentional risk-taking to impress their peers or significant others. As one patient described it, “*pushing boundaries”* is part of the young rural male rite of passage.

The third theme—labelled ‘hopelessness’—was only identified by Indigenous patients and is not unlike that reported by Finlayson and Auld [33]. In discussing the motivations underpinning risky behaviour, several Indigenous patients suggested that they had “given up”—like many in their community—and were not worried about safety because their life was hopeless, often linking excessive alcohol use with unemployment and being unlicensed. Non-Indigenous patients, on the other hand, were more likely to externalize blame. “Locus of Control” is a complex measure. In studies of young at-risk drivers, it has been found to have differing impact and to sometimes be related to unsafe driving and sometimes not [34,35]. 

In the present study locus of control appears in two ways with the Indigenous respondents. It is clearly personalised as a proximal influence on crash involvement by Indigenous respondents through their personal drinking and drug use. This contrasts with the non-Indigenous attribution of the cause of crashes to external factors such as animals, road conditions and bad luck. However, Indigenous respondents also raised social factors as distal influences that lay outside their control and raised limited police presence, lack of access to licensing and limited vehicle ownership. Both of the latter have a direct impact on pedestrian and passenger safety.

### 3.2. Quantitative Analysis

#### Results

From the themes identified in the above qualitative analysis of the narratives, seventeen variables of interest, derived from responses to the structured portion of the interview schedule, were considered for quantitative analysis. The first thirteen, to be analysed by both Indigenous status and remoteness are listed below—of which, the first nine were associated with established personal behaviours and the next four with the specific location. The remaining four, described below, are attitudinal and analysed by Indigenous status only without reference to remoteness of residence.

1. Habitual alcohol use and possible dependency—here, equated with harmful drinking.
2. Drink driving in the previous month, defined as driving after consuming two or more alcoholic drinks in the hour prior.
3. Being a passenger of a drink driver (the latter similarly defined) in the past month.
4. Illicit drug use in the 24 h prior to the crash.
5. Crash history in the previous five years.
6. Unlicensed at time of crash.
7. Unemployed at time of crash.
8. Self-reported general health.
9. Lower perceived locus of control—derived from responses to questions about the preventability of crashes [21] (pp.155).
10. Being distracted immediately before the crash.
11. Not using personal protective equipment (seat belt or helmet) at time of crash. (For a participant to be classed as a user, all occupants of the vehicle should have been compliant.)
12. Travelling above the speed limit at time of crash.
13. Fatigued at time of crash.

Four of the above variables (1,7–9), concerning habitual alcohol use, unemployment, health and perceived locus of control, are elements of distal causation; the remaining nine are elements of proximal causation and are defined as transport-related—that is, recent or past driving and passenger history, and substance use and other circumstances or activities in the period leading up to the crash.

The four additional attitudinal variables were participants’ concern for road safety; for employment; and for family; and to what extent they believed their traffic behaviour to be similar to others in their community. These were measured on a five-point scale but have been dichotomised as positive if the response was “lots” or “a fair bit”. Responses to these questions are presented only as percentages for Indigenous and non-Indigenous respondents separately without regard to their remoteness (Table 1).

Table 2 shows the profile of road user involvement in crashes for these two groups. Non-Indigenous participants are largely motor-vehicle controllers (76%) in contrast to the Indigenous participants (43%); 52% of the Indigenous participants and 20% of the non-Indigenous participants were passengers or pedestrians. The sample involved a high proportion of single vehicle crashes in both remote areas (84%) and rural areas (79%) as reported by participants.

In the case of variable (1), habitual alcohol use analysis of variance showed the mean AUDIT-C scores for the Indigenous sample, 6.7, to be appreciably higher than that for the non-Indigenous sample, 5.2, (*p* = 0.005). In addition, the mean score among all participants residing in remote locations was also notably higher, 6.4, than participants residing in rural locations, 5.1, (*p* = 0.05), with no evidence of an interaction. However, a closer examination of the distribution of AUDIT-C scores among the Indigenous sample shows that it is bimodal with a large proportion of abstainers and a similarly large proportion of heavy drinkers, whereas the distribution in the non-Indigenous sample is essentially unimodal. Taking the definition of harmful drinking (an AUDIT-C score of ≥ 4 for men and ≥ 3 for women) as proposed by Bush and colleagues [26] the proportions of harmful drinking among these Indigenous and non-Indigenous participants who have been involved in a road crash are 75% and 70%, respectively, and 77% and 68% among remote and rural residents, respectively. 

Analytic results for the dichotomous factors are presented in Table 3 for each Indigenous status/remoteness cell and for Indigenous status as a whole as positive responses upon total valid responses and derived percentage. To save space, totals have been presented for only the Indigenous status categories since the large rural non-Indigenous cell comprises over 90% of the rural category, effectively equivalent to the total.

Of the nine transport-related factors, five showed positive associations with Indigenous status:

Indigenous participants were more likely to have driven after drinking in the previous month (OR: 2.3, *p* = 0.03), to have been a passenger of a drink driver in the previous month (OR: 2.9, *p* = 0.005) and to have been distracted (OR: 2.8, *p* = 0.01) or fatigued (OR: 3.5, *p* = 0.003) before the crash, and more likely be unlicensed at the time of the crash (OR: 2.9, *p* = 0.025). Remoteness of residence was also associated with unlicensed driving at the time of the crash (OR: 2.8, *p* = 0.002), while participants residing in a remote area were more likely to have taken illicit drugs in the 24 hours beforehand (OR: 2.7, *p* = 0.01). Indigenous participants residing in a remote area were more likely to have been involved in a traffic crash in the previous five years (Interaction OR: 4.6, *p* = 0.02), whereas there was little difference by remoteness in non-Indigenous participants. There is also a suggestion that Indigenous participants crashing in a remote area were more likely to have failed to use personal protective equipment (OR: 2.2, *p* = 0.06). Speeding showed no association with either Indigenous status or remoteness.

Unemployment was more common among Indigenous participants (OR: 3.3, *p* = 0.005), in particular those residing in remote areas (Interaction OR: 5.5, *p* = 0.006). Perceived locus of control was weaker among Indigenous participants (OR: 2.6, *p* = 0.02). This may reflect the differences in the themes of the qualitative study where there were marked differences between the two groups in their perceptions of both proximal and distal causation of their crashes. Unlike the non-Indigenous participants, Indigenous participants residing in remote areas reported somewhat poorer health than non-Indigenous participants and Indigenous participants residing in rural areas (Interaction OR: 2.9, *p* = 0.06).

## 4. Discussion 

Through a thematic analysis of personal crash narratives, this research was able to provide an improved understanding from the participants’ perspective of Indigenous road crash experience compared with non-Indigenous respondents in the broader rural and remote context. These themes were further examined quantitatively using in addition respondents from the RRRSS to increase the power of the statistical analysis to determine the association of relevant themes with Indigenous status and remoteness.

The active participation and candid responses whereby participants “tell their story” that are reported in the present qualitative paper enables a more complete picture of the road user profile and behavioural contributors (proximal causation) to crashes. In addition, the methodology made it possible to explore the broader social context in which unsafe behaviours occur and influence the motivations for risk-taking (distal causation). 

In terms of crash causation, behavioural themes figured prominently in both Indigenous and non-Indigenous crash narratives: alcohol use was frequently mentioned and was more likely to be acknowledged as a contributing factor by the Indigenous respondents. Non-Indigenous participants were more likely to prioritise road characteristics or other external (non-personal) factors including just “being unlucky” as contributing to their crashes. Being distracted and being fatigued immediately before the crash were both commonly reported by both groups though the Indigenous respondents were more likely to attribute this to prior drinking activities. In the literature or in crash data, being fatigued at the time of crash has not been identified as a significant risk factor for Indigenous persons [11]. However, this may be due to a procedural factor, given that few follow-up questions are asked of crashing drivers after alcohol is assumed to be a primary causal factor following its detection via a quantifiable measure, such as breath or blood sample. Consequently, fatigue would go unreported in most cases. Both groups reported travelling above the speed limit at the time of crash and inappropriate speed for the conditions of the road. Often the crash involved a combination of these.

Another common theme reported by both groups was the failure to wear protective equipment such as seat belts and helmets and the high-risk behaviour of being a passenger in the back of service vehicles (“utes”). This has been identified as a major problem of Indigenous road safety [22]. Anecdotal evidence, such as newspaper reports, together with the quotes above suggests that this is a general problem in rural and remote regions. 

The examination of behaviour in its broader distal social context yielded both similarities and differences between Indigenous and non-Indigenous participants. The crash narratives highlighted two consistent motivations for both Indigenous and non-Indigenous participants: first, social acceptance of risk as part of the ‘rural way of life’; and also rural bravado through “being a hero” as a young male rite of passage. A third theme, described by Indigenous participants only, related to lower locus of control and feelings of hopelessness due to poor life circumstances (limited financial resources, boredom, unemployment) and “nobody to teach me the rules” which was associated with reduced access to licensing services thus elevating crash risk (distal causation). However, Indigenous respondents were more likely to take responsibility for their crashes than non-Indigenous drivers, suggesting that in respect of proximal causation perceived locus of control was higher in the Indigenous than in the non-Indigenous. This in turn implies that the locus of control is a somewhat more complex concept than perhaps generally recognised.

From a policy perspective, the challenge for safety is to move knowledge generated by this research into efforts to encourage positive behaviour change. In rural and remote settings, an Engineering, Enforcement and Education approach may prove more difficult from a resourcing perspective.

There is potential to work collaboratively across boundaries to influence behaviours more broadly. For example, in terms of enforcement, police authorities need to recognize the value of regional and network operational planning, as well as saturation of high-profile community events, as a better deterrence strategy, than the traditional district approach.

In terms of engineering, road authorities need to capitalize on cost sharing/joint purchasing arrangements to increase resources, thus getting better network coverage. Similarly, safety works allocations/prioritization on the rural network need to be aligned with prominent crash profiles (i.e., run off road on curve or straight and head-on crashes), as well as increased focus on speed management and aligning speed limits with road conditions.

The potential to improve behaviours by addressing social context (distal causation) through education and cross-agency mobilization provides the greatest opportunity to influence the rural and remote and Indigenous road toll moving forward. The benefits of directing programs to the needs and social context of Indigenous persons has been recently confirmed by the positive findings of the evaluation of a New Zealand drink driving rehabilitation programme specifically designed for Māori offenders [36]. 

One key way forward borne out of the research is the need to address perceived hopelessness among Indigenous people through improvements to the licensing process, leading to better life circumstances [14]. This requires integrating road safety into other policy agendas (employment) and building on community strengths and learning styles as an educational strategy.

In terms of study limitations, non-participation rates (those not approached or refusals) were not able to be obtained for both ethical and logistical reasons. Feedback was sought from representatives of participating facilities post-data collection on the methodology and research in general. In terms of process, interviewees were simply asked to articulate *“what worked”* and *“what didn’t work”*. Interviewees provided support for the methodology, highlighting that the assurance of confidentiality and story-telling interview style lead to a positive response from those approached. In particular they reported that most of those approached were eager to share their experiences anonymously, suggesting that outright refusals were uncommon. On the other hand, unfamiliarity with the project by new clinic staff or pressure of time had sometimes led to potential subjects being missed; this will have occurred independently of personal characteristics or circumstances of the traffic incident and cannot have been a source of bias. However, some clinic staff admitted that they had been more assiduous in promoting the study to persons they considered more culpable and in need of counselling [9] (pp. 209–211).

## 5. Conclusions

While many of the familiar risk factors for road crashes and associated injuries—alcohol use, speeding, non-use of restraints, fatigue and distraction—were common to both Indigenous and non-Indigenous groups, they tended to be more prevalent among Indigenous crashes and more prevalent for both groups in remote areas. Indigenous respondents were more likely to accept responsibility for the actions which lead to the crash. Non-Indigenous respondents tended to lay the blame on outside factors such as road quality or animals on the road. They were also more likely to place higher value on road and personal safety. Indigenous respondents demonstrated more concern over employment or family safety, consistent with the feelings of hopelessness which came through from the crash narratives of many Indigenous respondents, and as reported elsewhere.

It was clear that the conventional messages on road safety directed at the general Australian public have little meaning for Indigenous communities in rural and remote areas. Efforts to promote road safety in such communities need to go hand in hand with steps to ameliorate their life circumstances. Given the high rate of passenger and pedestrian road crash injuries comparatively, an increased focus on developing safety messaging for these road user groups should be a priority, extending the current focus on driver-based education.

There are many regions in Australia similar to North Queensland, with both rural and remote areas and a high proportion of Indigenous inhabitants, to which our findings and recommendations apply.

## Figures and Tables

**Table 1:** Attitudinal comparisons by Indigenous status.

Attitude	Indigenous	Non-Indigenous
Concern re: employment	72 of 80 (90%)	50 of 147 (34%)
Concern re: road safety	40 of 80 (50%)	91 of 147 (62%)
Worry re: personal safety	35 of 79 (44%)	104 of 147 (71%)
Worry re: family safety	59 of 79 (75%)	90 of 147 (61%)
Same as my community	58 of 80 (73%)	70 of 147 (48%)

**Table 2:** Road user types by Indigenous status.

	Indigenous		Non-Indigenous	
Road User Type	*N*	%	*N*	%
Driver	42	36.8	167	33.0
Rider	7	6.1	220	43.4
Passenger	45	39.5	89	17.6
Pedestrian	14	12.3	11	2.2
Cyclist	6	5.3	19	3.8
Total	114	100.0	506	100.0

**Table 3:** (**A**) Results from analysis of the dichotomous variables. (**B**) Logistic Regression.

**(c) Passenger of drink driver in previous month**						
**Variables**	**Remote**	**%**	**Rural**	**%**	**Total**	**%**
Indigenous	60/80	75.0	15/33	45.5	75/113	66.4
Non-Indigenous	25/87	28.7	72/352	22.6	97/406	23.9
**Logistic Regression**	**χ^2^**	***p***	**OR**			
Indigenous status	7.87	0.005	2.9			
Remoteness	1.42	0.23	1.4			
**(d) Illicit drug use in the 24 hours prior to the crash**						
**Variables**	**Remote**	**%**	**Rural**	**%**	**Total**	**%**
Non-Indigenous	12/89	13.5	17/313	5.4	29/402	7.2
**Logistic Regression**	**χ^2^**	***p***	**OR**			
Indigenous status	0.72	0.40	1.7			
Remoteness	6.29	0.012	2.7			
**(e) Crash history in the previous five years**						
**Variables**	**Remote**	**%**	**Rural**	**%**	**Total**	**%**
Non-Indigenous	18/91	19.8	78/324	24.1	96/415	23.1
**Logistic Regression**	**χ^2^**	***p***	**OR**			
Indigenous status	2.50	0.114	0.4			
Remoteness	0.73	0.39	0.8			
Interaction	5.53	0.02	4.6			
**(f) Unlicensed at time of crash**						
**Variables**	**Remote**	**%**	**Rural**	**%**	**Total**	**%**
Non-Indigenous	19/78	24.4	27/266	10.2	45/344	13.4
**Logistic Regression**	**χ^2^**	***p***	**OR**			
Indigenous status	5.05	0.025	2.9			
Remoteness	9.90	0.002	2.8			
**(g) Unemployed at time of crash**						
**Variables**	**Remote**	**%**	**Rural**	**%**	**Total**	**%**
Non-Indigenous	7/86	8.1	34/289	11.8	41/375	10.9
**Logistic Regression**	**χ^2^**	***p***	**OR**			
Indigenous status	7.90	0.005	3.3			
Remoteness	0.88	0.35	1.5			
Interaction	7.58	0.006	5.5			
**(h) Self-reportedly generally healthy**						
**Variables**	**Remote**	**%**	**Rural**	**%**	**Total**	**%**
Non-Indigenous	75/88	85.2	267/312	84.2	342/405	84.4
**Logistic Regression**	**χ^2^**	***p***	**OR**			
Indigenous status	1.32	0.25	1.6			
Remoteness	0.05	0.82	0.9			
Interaction	3.54	0.06	2.9			
**(i) Lower perceived locus of control**						
**Variables**	**Remote**	**%**	**Rural**	**%**	**Total**	**%**
Non-Indigenous	52/90	57.8	168/326	51.5	220/416	52.9
**Logistic Regression**	**χ^2^**	***p***	**OR**			
Indigenous status	5.64	0.018	2.6			
Remoteness	1.10	0.29	1.3			
**(j) Distraction before crash**						
**Variables**	**Remote**	**%**	**Rural**	**%**	**Total**	**%**
Non-Indigenous	56/167	33.5	120/306	39.2	176/473	37.2
**Logistic Regression**	**χ^2^**	***p***	**OR**			
Indigenous status	6.94	0.008	2.8			
Remoteness	1.49	0.22	0.8			
**(k) Failure to use personal protective equipment at time of crash**						
**Variables**	**Remote**	**%**	**Rural**	**%**	**Total**	**%**
Non-Indigenous	37/157	23.6	57/311	18.3	94/468	20.1
**Logistic Regression**	**χ^2^**	***p***	**OR**			
Indigenous status	3.41	0.065	2.2			
Remoteness	1.78	0.18	1.4			
**(l) Travelling above the speed limit at time of crash**						
**Variables**	**Remote**	**%**	**Rural**	**%**	**Total**	**%**
Non-Indigenous	19/140	13.6	34/280	12.1	53/420	12.6
**Logistic Regression**	**χ^2^**	***p***	**OR**			
Indigenous status	0.89	0.35	1.6			
Remoteness	0.17	0.68				
**(m) Fatigued at time of crash**						
**Variables**	**Remote**	**%**	**Rural**	**%**	**Total**	**%**
Non-Indigenous	17/149	11.4	42/269	15.6	59/418	14.1
**Logistic Regression**	**χ^2^**	***p***	**OR**			
Indigenous status	8.81	0.003	3.5			
Remoteness	1.39	0.24	0.7			
